# Pervasive Defaunation of Forest Remnants in a Tropical Biodiversity Hotspot

**DOI:** 10.1371/journal.pone.0041671

**Published:** 2012-08-14

**Authors:** Gustavo R. Canale, Carlos A. Peres, Carlos E. Guidorizzi, Cassiano A. Ferreira Gatto, Maria Cecília M. Kierulff

**Affiliations:** 1 Wildlife Research Group, Anatomy School, University of Cambridge, Cambridge, United Kingdom; 2 Department of Biology, Universidade do Estado de Mato Grosso (UNEMAT), Tangará da Serra, Mato Grosso, Brazil; 3 Centre for Biodiversity Research, School of Environmental Sciences, University of East Anglia, Norwich, United Kingdom; 4 Instituto Chico Mendes, Brazilian Ministry of Environment ICMBIO, Brasilia DF, Brazil; 5 Coordenação de Ecologia, Instituto Nacional de Pesquisa da Amazonia (INPA), Manaus, Amazonas, Brazil; 6 Instituto Pri-Matas para a Conservação da Biodiversidade, Belo Horizonte, Minas Gerais, Brazil; Centre National de la Recherche Scientifique, France

## Abstract

Tropical deforestation and forest fragmentation are among the most important biodiversity conservation issues worldwide, yet local extinctions of millions of animal and plant populations stranded in unprotected forest remnants remain poorly explained. Here, we report unprecedented rates of local extinctions of medium to large-bodied mammals in one of the world's most important tropical biodiversity hotspots. We scrutinized 8,846 person-years of local knowledge to derive patch occupancy data for 18 mammal species within 196 forest patches across a 252,669-km^2^ study region of the Brazilian Atlantic Forest. We uncovered a staggering rate of local extinctions in the mammal fauna, with only 767 from a possible 3,528 populations still persisting. On average, forest patches retained 3.9 out of 18 potential species occupancies, and geographic ranges had contracted to 0–14.4% of their former distributions, including five large-bodied species that had been extirpated at a regional scale. Forest fragments were highly accessible to hunters and exposed to edge effects and fires, thereby severely diminishing the predictive power of species-area relationships, with the power model explaining only ∼9% of the variation in species richness per patch. Hence, conventional species-area curves provided over-optimistic estimates of species persistence in that most forest fragments had lost species at a much faster rate than predicted by habitat loss alone.

## Introduction

Tropical deforestation will continue to occupy the center of most 21^st^-century conservation agendas due to its disproportionate role in local to regional scale extinctions in terrestrial ecosystems worldwide [1], [2]. The consequences of both habitat loss and fragmentation to population extinctions in tropical forest landscapes remain poorly understood and largely based on unqualified theoretical conjectures derived from the species-area relationship [3], [4]. In practice, tropical forest remnants persisting in real-world human-dominated landscapes coexist with a range of other forms of anthropogenic perturbations, including overhunting, timber extraction, surface fires, invasion of exotic species, and mesopredator release via removal of top-down control [5]–[7]. These secondary impacts can interact synergistically with the twin effects of reduced habitat area and increased isolation, thereby potentially exacerbating the rate of biodiversity loss within remaining forest fragments.

**Figure 1:**
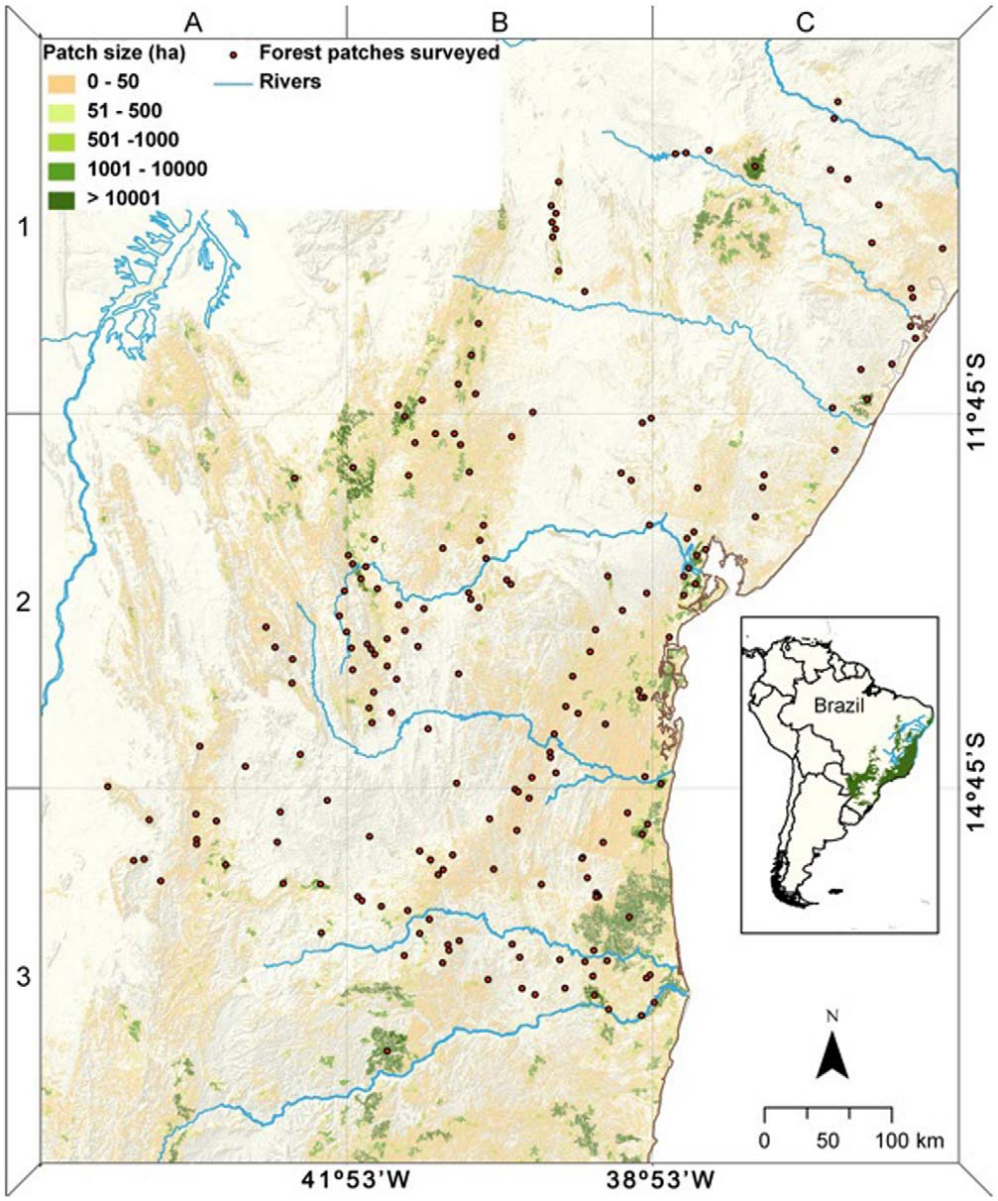
Distribution of remaining forest patches across the northern Atlantic Forest study region showing all surveyed forest patches. For detailed maps see Fig. S1, S2, S3.

**Figure 2:**
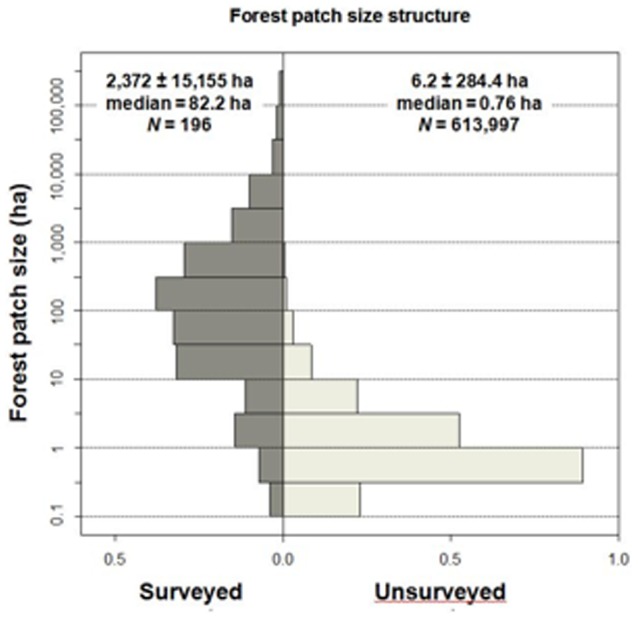
Size distribution of all forest patches surveyed (left, *N* = 196) or not surveyed (right, *N* = 613,997 patches >0.25 ha) within the entire study region (∼252,669 km^2^). Numerical representation of different-sized patches are expressed in terms of proportions of the total.

The once vast Atlantic Forest biome (∼1.5 million km^2^) is one of the ‘hottest’ of the global biodiversity hotspots [8] due to unrivalled numbers of endemic species and a relentless process of post-colonial deforestation. Currently, ∼89.2% of the original forest cover (∼37,365,280 ha) of the four biogeographic subregions (BSRs) of the Atlantic Forest surveyed in this study have been converted to other land-uses [9] and the remaining forest cover is heavily skewed to very small fragments (<10 ha) (Fig. 1 and 2). Within the entire biome, the Bahia Centre of Endemism is often considered to be one of the most species-rich, retaining the highest levels of local endemism [10], which is partly attributed to its stable forest refugia during late Pleistocene climatic extremes [11]. Although this BSR has a long history of deforestation following 16^th^-century European conquest [12], [13], ∼75% of its forest cover persisted until 1945, with deforestation in the mid 1970s spreading rapidly throughout the region following a peak of road-building [14].

Here, we quantify local extinction rates for 10 terrestrial and 7 arboreal midsized to large-bodied mammal species across a large sample of forest patches remaining within a once entirely forested region, encompassing 25,266,865 ha of four BSRs of the Atlantic Forest of northeastern Brazil. We also examine patch occupancy of a widely known small-bodied species to further assess the reliability of local interviews. We compare these empirical estimates to predictions based entirely on the species-area relationship (SAR), which as a power model is a straight-line in log-log space where the slope is the z-value [*S*_new_/*S*_original_ = (*A*_new_/*A*_original_)^z^] [15]. We selected a widely scattered set of 196 variable-sized forest fragments (range = 0.17–194,341 ha) (Table S1) from the nearly 738,887 remaining forest patches available (Fig. 1) across the region on the basis of recent Landsat images, which were used to calculate both patch and landscape metrics, and relate these to mammal species richness and composition. We also explicitly consider the role of human population density and the history of anthropogenic disturbance within and around each surveyed fragment in determining local extinctions. Finally, we estimate the degree to which the geographic range of these forest species had effectively contracted on a regional scale in modern times. To our knowledge, this is the first attempt to estimate local extinction rates of midsized and large mammals and model predictors of extirpation at a regional scale. This study is also the first to document the spatial extent of extirpation of five large neotropical forest mammal species (jaguar, lowland tapir, white-lipped peccaries, woolly spider-monkey and giant anteater) throughout the Atlantic Forest biome.

## Results

### (a) Patterns of Regional-Scale Extinctions

Even the most intact forest fragments remaining across this vast study region had lost some of their midsized and large-bodied mammal fauna, and none of the sites surveyed had a full complement of species. Of the maximum number of 3,528 populations (18 species at 196 sites) that once occupied our entire forest region, only 767 (21.7%) were still extant, regardless of their level of demographic and genetic viability. These results are in contrast with far more optimistic predictions from classic species-area relationships (SARs) [15], which would forecast an overall persistence rate of 46.8% to 82.7% of all mammal populations for the fragments surveyed on the basis of reasonable bounds of *z*-values ranging from 0.4 to 0.1 [16], and the total proportional forest area remaining across the four BSRs that we surveyed (15.02%, Ref. 9). However, applying SARs derived from these 196 surveyed fragments to all 738,887 fragments remaining across the study region (Fig. 2) would yield an even lower overall population persistence rate of 15.1% (predicted total of 2,008,295 from a maximum of 13,299,966 populations).

**Figure 3:**
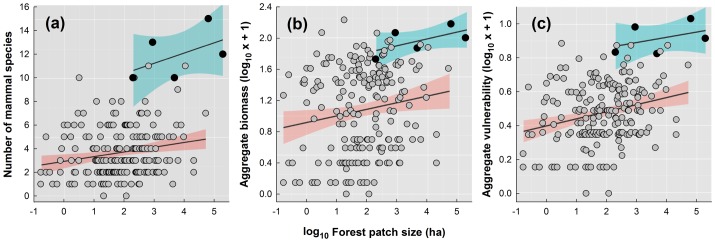
Species-area relationships (SARs) for (a) species richness, (b) a measure of total biomass, and (c) a measure of total species vulnerability for 18 mammal species surveyed at 196 forest patches of the Atlantic forest of northeastern Brazil. Solid circles (and corresponding regression lines and 95% confidence intervals) indicate the five existing strictly protected forest areas in the entire study region, for which intercepts were clearly higher. All other data points (gray circles) represent unprotected forest sites.

Both small and large forest remnants varied widely in the number of species they retained. On average, only 3.3 (±2.1 SD) of the 18 species sampled persisted in patches smaller than 50 ha, which vastly dominated the remaining forest cover in the study region (Fig. 2). This mean patch-scale persistence rate increased only moderately to 5.0 (±3.3) and 4.0 (±2.1) species in larger forest patches of 500–1,000 ha and 1,000–5,000 ha, respectively (Table S2). Even fragments larger than 5,000 ha on average harbored only 7.2 species (±4.1), further confirming that high rates of local extinctions were widespread even in the largest forest patches located within the former ranges of the 18 mammal species. Contrary to most empirical SARs for tropical vertebrates, forest patch area alone explained only a small fraction of the variation in mammal species richness (R^2^_adj_ = 9.4%; Fig. 3), and none of the eight SAR models in the literature (Text S1) provided an adequate fit to the transformed or untransformed data. Likewise, patch area alone failed to predict most of the variation in a measure of aggregate mammal biomass (R^2^_adj_ = 4.4%; Fig. 3) and aggregate mammal vulnerability of the species persisting in each forest patch (R^2^_adj_ = 10.2%; Fig. 3). On a positive note, however, the five protected areas established throughout the study region substantially elevated overall species persistence: 5 of the 9 sites retaining 10 or more species were strictly protected forest reserves. Protected areas on average retained over three times as many species, and those species were on average 1.5-fold larger and shared a species vulnerability index 3.4-fold higher than those in unprotected areas (Walsh's approximation unequal variance t-tests, p<0.02 in all cases; Fig. 3).

### (b) Patterns of Local Extinctions

**Figure 4:**
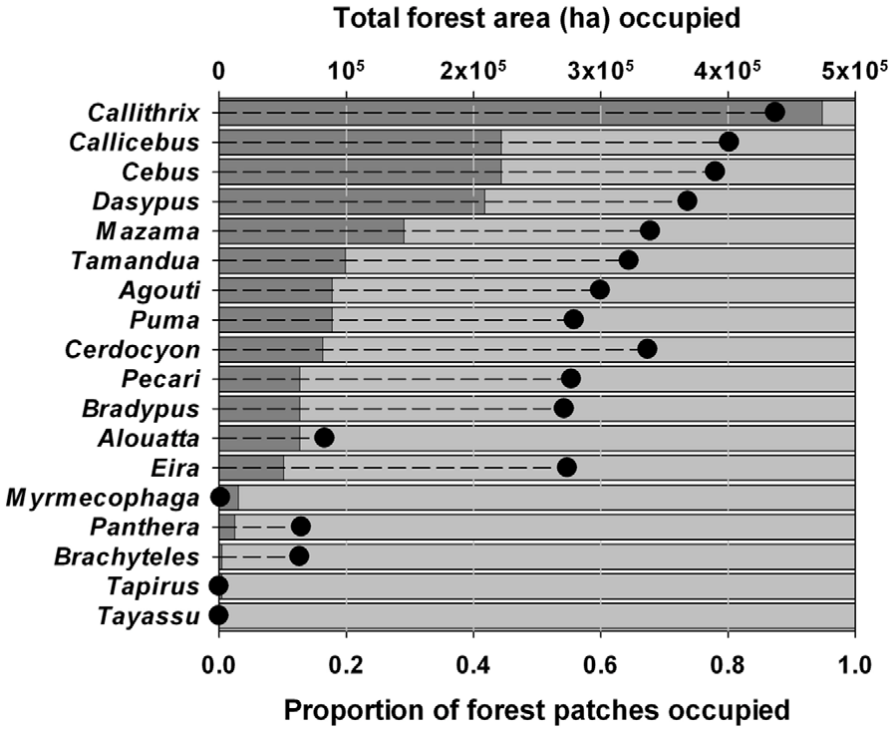
Levels of forest patch occupancy for 18 mammal species surveyed throughout the Atlantic Forest study region of northeastern Brazil. Species are ordered top to bottom according to increasing level of patch occupancy. Dark-gray bars indicate the proportion of all 196 forest patches occupied by each species (see Table 1 for full Latin and English names), and solid circles indicate the aggregate forest area contained within all occupied patches.

On average, forest patches contained only 3.9±2.5 species [range = 0–15], but most of these were small to midsized, disturbance-tolerant species, such as small primates (marmosets and titi monkeys) and nine-banded armadillos, that were still ubiquitous throughout the survey region (Table 1; Fig. 4). Of the 18 species sampled, only the smallest species (marmosets) persisted in over half of all 196 forest patches. Three species occupied less than 1% of the combined area of all surveyed patches (Table 1; Fig. 4). Eleven of the 18 species sampled are larger than 5 kg and these fared far worse, with only 230 (10.7%) from a maximum of 2,156 populations (11 species at 196 sites) still persisting. White-lipped peccaries had been completely extirpated throughout the entire region, and four of the other seven large-bodied species (>9.5 kg) (lowland tapir, woolly-spider monkey, jaguar and giant anteater) nearly so, occurring in only 1 to 6 of the 196 patches (Table 1; Fig. 4). On the basis of the minimum observed forest patch size where each species were known to occur, we conservatively scaled up these local extinctions to all 738,887 forest fragments remaining across the entire ∼25.3×10^6^ ha study region. This translates into dramatic contractions in the remaining effective range size of all species, with only the smallest-bodied species occurring in ∼14% of their former geographic range (Table 1). Eleven of 12 species larger than 5 kg were restricted to only <3% of the entire region. Moreover, these extinctions were often reported to pre-date most of the historical deforestation and land-use change across the region; for instance, interviews clearly indicated that there was no record in recent memory of these otherwise widespread species in all but a few forest fragments surveyed. Only white-lipped peccary and woolly-spider monkey were reported to have been locally extirpated in the last decade prior to interviews (but in only 2 and 3 patches, respectively) and the largest five species were reported by 98% of all interviewees to have disappeared from local folklore, and were no longer pursued by subsistence hunters as viable game species.

**Table 1:** Body mass, forest patch occupancy (N = 196 forest fragments) and regional scale estimates of geographic range contraction for the 18 mammal species surveyed in this study.

Species	English name	Body Mass (kg)	Patch occupancy (%)	Smallest patch (ha)	% Total area of occupied patches	% Geographic range occupied (252,668 km^2^)	Max. area occupied (ha)‡	Total area likely to be occupied (ha)*	% Area likely to be occupied†
*Callithrix jacchus/penicillata/kuhlii*	Common marmoset/Black-tufted marmoset/Kuhl's marmoset	0.4	95.41	0.17	98.70	14.39	3,811,641	3,636,616	95.41
*Callicebus melanochir/barbarabrownae*	Coastal black-handed titi/Barbara Brown's titi monkey	1.1	44.90	0.25	90.92	6.74	3,790,634	1,701,917	44.65
*Cebus xanthosternos*	Yellow-breasted capuchin monkey	2.5	44.39	0.76	84.05	6.45	3,672,727	1,630,241	42.77
*Alouatta guariba/caraya*	Brown howler/Black howler monkey	6.5	12.76	2.19	17.87	1.74	3,447,391	439,718	11.54
*Brachyteles hypoxanthus*	Woolly-spider monkey	9.5	0.51	63441.93	13.64	0.01	257,783	1,315	0.03
*Bradypus torquatus/variegatus*	Maned sloth/Three-toed sloth	5.0	13.78	1.01	60.85	1.97	3,622,006	498,950	13.09
*Myrmecophaga tridactyla*	Giant anteater	15.0	3.06	3.78	0.28	0.40	3,293,997	100,837	2.65
*Tamandua tetradactyla*	Collared anteater	4.5	20.92	0.84	71.81	3.03	3,655,035	764,574	20.06
*Dasypus novemcinctus*	Nine-banded armadillo	3.3	43.37	0.25	86.25	6.51	3,790,634	1,643,897	43.13
*Agouti paca*	Paca	9.0	18.88	1.01	66.95	2.71	3,622,006	683,746	17.94
*Puma concolor*	Puma	25.0	17.86	1.35	60.02	2.52	3,563,450	636,330	16.69
*Panthera onca*	Jaguar	35.0	2.55	3.20	13.93	0.34	3,343,534	85,294	2.24
*Cerdocyon thous*	Crab-eating fox	6.0	17.35	0.25	74.98	2.60	3,790,634	657,559	17.25
*Eira barbara*	Tayra	4.0	11.22	1.01	61.39	1.61	3,622,006	406,552	10.66
*Mazama spp.*	Brocket deer	22.0	30.10	0.25	75.44	4.52	3,790,634	1,141,058	29.94
*Pecari tajacu*	Collared peccary	17.0	13.78	0.17	62.08	2.08	3,811,641	525,073	13.78
*Tayassu pecari*	White-lipped peccary	28.0	-	-	-	0.00	-	-	0.00
*Tapirus terrestris*	Lowland tapir	150.0	0.51	17.66	0.003	0.06	2,765,624	14,110	0.37

‡ Total area of fragments larger than the smallest occupied patch throughout the entire study region, on the basis of 738,887 fragments >0.1 ha encompassing ∼3.8 million ha of forest.

* Aggregate area of forest fragments larger than the smallest occupied patch on the basis of patch occupancy data.

† Percentage of total area likely to be occupied in relation to the total area of forest fragments within the entire study region (∼3.8 million ha).

### (c) Environmental Predictors of Extinctions

Forest protection status and patch area were the only predictors consistently appearing in all best candidate models explaining contemporary species richness, but the importance of protection was consistently greater than patch size in terms of both species persistence and key traits that predispose species to local extinctions such as body mass, hunter preference and per capita fecundity (Tables S3 and S4). Most other patch and landscape variables were relatively unimportant, with the exception of topographic slope, whereby steeply dissected terrains — that are less physically accessible to both present and past hunters — boosted persistence of extant populations, particularly large-bodied, vulnerable taxa. Higher-elevation sites were also more likely to retain large-bodied species. Surprisingly, contemporary household density had only a modest effect on species persistence, but this can be attributed to considerable changes in the size and spatial distribution of the human population in this region since most local extinctions occurred. Likewise, measures of landscape connectivity including inter-patch proximity and the proportion of forest cover remaining within matrix areas had a marginal effect on levels of species persistence, likely because source populations in the neighborhood of fragments were no longer available regardless of the extent to which the intervening matrix was permeable.

## Discussion

Second to the late-Pleistocene Nearctic-Neotropical megafaunal overkill [17], our findings show the staggering rate of human-induced eradication of the modern midsized to large-bodied mammal fauna across a vast region of one of the world's most important tropical forest biodiversity hotspots. That these remnant forest areas of the Atlantic Forest of northeastern Brazil have been heavily depleted of their charismatic mammals is further stressed by the fact that the 196 patches that we surveyed represented a carefully-selected sample of the most intact and best preserved forest remnants left in this entire region; most unsurveyed fragments are therefore expected to retain far more depauperate mammal assemblages and smaller populations given that they are orders of magnitude smaller and considerably more degraded (in their forest structure and floristic composition) than those that we did survey. For example, a crude extrapolation of the SAR derived from this study to the 738,887 forest fragments >0.1 ha remaining across our study region results in an overall extinction rate of ∼85% of all mammal populations. This scenario is confirmed by observations elsewhere in the northern Atlantic Forest, where fewer than 50% of all midsized and large mammals persist in fragments <500 ha [18]. This is surprising given that several of the large mammals we surveyed are relatively matrix-tolerant and able to persist in many human-modified landscapes [19], [20], suggesting that any source populations in the surrounding landscape had also been depleted.

Only three primate taxa (*Callithrix* spp., *Callicebus* spp. and *Cebus xanthosternos*) and nine-banded armadillos (*Dasypus novemcinctus*) could be defined as sufficiently resilient to persist in >40% of the forest patches surveyed (Fig. 4 and Tab. 1). Other species that typically persist in small but well protected forest patches elsewhere, such as woolly-spider monkeys [21], howler monkeys [22] and common sloths [23], were likely driven to local extinction by overhunting in most remnants. Jaguars and pumas were not hunted for food, but were often persecuted and killed because they were perceived as threats to humans and livestock. The largest game species were virtually extinct across the entire study region, as they often require sustainable offtake areas >2,000 km^2^ [6], which are no longer available. Forest patches with a repeated post-isolation history of disturbance tended to retain only few generalist and highly matrix-tolerant species (e.g. *Cerdocyon thous* and *Pecari tajacu*), but even these taxa were missing from most overhunted sites (Fig. 4).

Athough habitat loss in global biodiversity hotspots may overestimate extinction rates [4], [24], our study shows that model projections based on naïve species-area curves can be hugely optimistic in the real-world. Habitat loss alone may be a good predictor of extinctions of threatened and endemic species in biodiversity hotspots, but this takes no account of pervasive synergistic effects of hunting, wildfires and other anthropogenic impacts on isolated populations which may lead to much higher extinction rates compared to predictions from unqualified SAR models alone. Overhunting inflates the vulnerability of game populations stranded in forest fragments, further aggravating the risk of local extinctions [6]. For example, applying to our study region a species-area curve (R^2^ = 0.634) — based on the same set of mammal species (but replacing *Brachyteles* for *Ateles*) — that was empirically-derived at a largely non-hunted fragmented landscape in southern Brazilian Amazonia [19], resulted in the overall extinction of only 35.8% of all populations, compared to 78.3% documented here. The extinction rates we recorded may be very high compared to theoretical SAR model predictions, but are still conservative given that many more small populations will be driven to extinction if further habitat loss and overhunting continues unabated. Nonlinear dynamics, threshold effects and surprise are common in highly modified systems due to cumulative human impacts, trophic cascades and stochastic effects [25]. Furthermore, additional extinctions of small populations are eventually expected over time in many forest patches resulting in a net loss in the number of species present before full post-relaxation conditions can be attained [26], [27].

In highly-fragmented landscapes reeling from the combined effects of pervasive habitat loss, patch-scale disturbance and overexploitation can vastly override the effects of patch size and isolation on species retention. Our study therefore illustrates the crucial conservation role of formal strictly protected areas, which cannot be replaced by government-mandated forest set-asides within private landholdings as required by Brazilian legislation. Alarmingly, most Atlantic Forest vertebrate species are restricted to formally unprotected lowland and lower montane forest fragments in private properties [28]. Mammal species persistence was clearly highest in the five strictly protected areas that we sampled, regardless of their size and landscape context. These no-take forest reserves also retained the largest-bodied and most vulnerable mammal species (Fig. 3). Yet only 1.7% of the remaining Atlantic Forest cover is strictly protected [9]. This scenario is far worse in the Atlantic Forest of northeastern Brazil where the total protected lowland forest cover is much smaller than in other BSRs farther south that still retain large high-elevation areas of low agricultural value [9], [10]. Considering the bias in mammal research effort towards the southern Atlantic Forest [29], a renewed focus on hitherto poorly sampled areas will contribute most to our understanding of patterns of biodiversity distribution and threats. Finally, this study confirms that setting aside structurally suitable forest habitat alone cannot guarantee the long-term persistence of the large vertebrate fauna in most tropical forest regions, which can only be achieved by protecting all constituent parts of forest ecosystems.

## Materials and Methods

### Ethics statement

All field workers in this study are Brazilian citizens who are both affiliated with Brazilian research institutions and completely aware of the ethics regulations in Brazilian federal and state-level legislation in relation to local interviews in rural parts of northeastern Brazil, where the study was conducted. Prior to the onset of the project, we had obtained the appropriate agreement from the Ministry of Environment (MMA), which funded part of this work, to deploy our field campaigns. All field activities, including wildlife population surveys and local interviews, were therefore conducted under the full rigor of Brazilian legislation (Law No. 5,197 of 3rd January 1967 (see http://www.planalto.gov.br/ccivil_03/leis/L5197.htm). Verbal permission was obtained from landowners/managers for wildlife population surveys, and we made every effort to ascertain that all face-to-face interviewees were willing informants for whom special ethics procedures in terms of property rights of “traditional indigenous knowledge” do not apply. In order to conduct interviews in small households and national parks we were only required to have a verbal agreement by landowners or managers of protected areas, all of whom had been contacted by phone/email or met personally prior to the onset of interviews. We are therefore satisfied that all field activities conducted as part of this study were legitimate, even considering the most rigorous interpretation of Brazilian law regarding the acquisition and use of field data that does not involve scientific specimens.

In Brazilian law, local non-indigenous communities and/or households can be approached without prior ethics approval provided that none of the procedures and/or methods involve any form of coercion, which in this study was never the case. Interviewees were not financially compensated for those interviews, which often only lasted 20–30 min. Furthermore, there were no signs of resentment following interviews from any of our informants. We therefore conclude that these interviews were conducted lawfully both within and outside the protected areas we visited.

All interviewers were sensitive of the often harsh local realities faced by smallholders in this part of Brazil, and our interviews were conducted in a socially benign context without the use of translators. Many of our informants were illiterate or semi-literate, yet our verbal face-to-face communication and exchanges with them were clearly unambiguous, and there were no misunderstandings as to the purpose of the interviews or the destination of any data gathered therein. Moreover, in all cases we guaranteed full anonymity of our interviewees to protect their privacy or any legal (mis)interpretation of wrongdoing on their part, particularly in relation to previous hunting activities in either protected or unprotected areas. We are therefore content with the way in which these interviews were conducted, and this is vindicated by the fact that we remain completely unaware of any (formal or informal) complaints or other forms of discontent in relation to the way in which local informants were approached.

### Field Interviews

We used 30-m resolution satellite images (Landsat 7 ETM, 2001, UTM, Datum Córrego Alegre) to pinpoint and select 196 forest fragments across a study region of ∼252,669 km^2^ encompassing portions of the Brazilian states of Bahia, Sergipe and Minas Gerais. This region included four of the eight recognized BSRs of the Brazilian Atlantic Forest biome [29]: Bahia (*N* = 141 sites), Diamantina (*N* = 37), São Francisco (*N* = 17), and Pernambuco (*N* = 1). Subsequently, we travelled a total distance of >205,000 km on paved and unpaved roads between January 2003 and January 2005 to survey individual fragments and interview a select set of 408 local residents to obtain patch-scale occupancy data on 18 medium to large-bodied mammal species within these fragments (Text S1). Because all mammal species addressed in this study are ubiquitous in forest habitat [29], they were almost certainly once widespread across the original forest cover throughout the study region. Recorded absences can therefore be interpreted as recent or historical local extinctions. Because these fragments were selected to maximize their size-spectrum and degree of forest structure integrity, they were considerably larger and in better structural condition than the total pool of 738,887 forest remnants (>0.1 ha) persisting across the entire region (Fig. 1), ∼85% of which are smaller than 10 ha (Fig. 2).

All 18 terrestrial and arboreal species considered in this study are coarse-scale forest habitat generalists, in that they occur within the Brazilian Atlantic Forest right across all forest types within the elevation range that we considered here, wherever extant populations persist today. These species were partly selected for the interviews and surveys because of their low level of forest microhabitat specificity. Furthermore, historical records of their geographic occurrence also show that they were widely distributed throughout the study region, and several species still occur well outside the typical forest domain of the Atlantic Forest biome, into more seasonally-dry transitional areas in the neighboring *Cerrado* and *Caatinga* domains. These species were therefore widespread right across the study region, and this is particularly the case of large-bodied, wide-ranging ‘landscape’ species that integrate large areas consisting of slightly different forest types. Our collective knowledge was combined with both IUCN maps and expert information obtained from other mammalogists working in the region to further ascertain that all forest fragments targeted in this study were indeed well within the known historical distributional range of the 18 species.

Interviewees were only selected during visits to each forest fragment if they were very familiar with the large mammal fauna and interviews were aided by color plates and high-quality photos, and in some cases playbacks of vocalizations, of each focal species to maximize accuracy in patch occupancy records. Additionally, color plates of five Amazonian and Mesoamerican mammal species (within the same spectrum of body mass as those surveyed here) *known not to occur* in the study region were also presented to each interviewee to quantify the prevalence of type II errors in the interview data. Further methodological details on forest patch selection, semi-structured interviews, field surveys, variable definition and data analysis are presented in Text S1.

Patch occupancy records of any given species were defined as patch-level incidence when interviewees had no doubt as to whether the species was locally present at the time of interviews or within 12 months prior to interviews, whether the species was thought to be a full-time resident or an occasional transient within that patch. Local extinction events were conservatively defined as unambiguous absences when a species had been reported to have once occurred within a patch but had not been sighted, heard or otherwise detected through tracks, scats, scrapes, burrows, vocalizations, or any other signs of their presence for at least three years. When compiling conflicting information from more than one interviewee referring to the same forest patch, we recorded a species as present if it had been unambiguously reported by at least one independent interviewee even if it had not been reported or otherwise omitted by another. We also excluded from the analysis other forest patches visited during the field campaigns (not shown in Figures S1, S2, S3) for which any of the 18 target species considered here was not reliably known to be either present or absent. Finally, this interview-based presence/absence sampling protocol has been successfully validated at 145 forest fragments elsewhere by four independent field sampling techniques, including diurnal line-transect censuses, camera trapping, intensive monitoring of sand track-plates, and counts of armadillo burrows [30]–[34].

### Forest patches attributes

We used 23 Landsat 7 ETM scenes (2001) to quantify key patch metrics (Area, Core Area, Perimeter, Gyrate and Shape indices) and landscape metrics (e.g. patch proximity and proportion of forest within 1-km external buffers) for all 196 forest patches surveyed, using Fragstats v. 3.3 coupled with ArcGIS 9.0. In addition, we calculated the percentage of total old-growth (primary) and successional forest cover (secondary forest older than ∼6 years) within a 1-km buffer area immediately outside the boundaries of the each forest patch. A total of 127 high-resolution digital maps (1∶100,000), which were obtained from the Brazilian Institute of Geography and Statistics (IBGE), were overlaid with the Landsat 7 ETM images to quantify topographic relief and the number of 5-m elevation contour lines (m above sea level) defining the steepness of the terrain of each patch. To verify patch-scale gradients in Δ-elevation we also extracted the minimum and maximum elevation for each forest fragment from four SRTM radar images (tiles 28/13, 28/14, 29/13, 29/14) downloaded from ***srtm*** *.csi.* ***cgiar*** *.org/*. We built elevation maps from SRTM images creating TIN (triangular irregular network) images with 30-m contour lines using the 3D Analyst tool from Arctool. We also measured the straight-line distance from each patch to both the nearest river or major perennial stream and paved/unpaved road used by motor vehicles. Based on local interviews and the high-resolution digital maps, we also recorded the total number of active rural households inside each patch and within a 2-km outside buffer zone from their perimeter to estimate the mean internal and external household density. Topographic slope and distance to roads and rivers were considered to be good proxies of the degree to which forest patches were physically accessible to extractive activities, including subsistence and commercial hunting; household density was considered as a good proxy of the degree to which this potential access could be realized; and forest protection status was a good proxy of the degree to which patches were legally accessible to hunters. All of these data were initially compiled using the best available databases, images, digital/hardcopy maps, and the aggregate information obtained from local informants, all of which were subsequently ground-truthed and verified during site visits (Text S1).

### Data Analysis

We considered a wide spectrum of explanatory variables describing the history of forest disturbance, physical accessibility, human population density, and patch and landscape variables associated with each forest isolate sampled. Based on generalized linear models (GLMs) followed by an AIC-based model averaging approach, we assess the relative importance of predictors of three assemblage-wide response variables: total species richness, aggregate body mass; and a measure of vulnerability of the mammal species retained in each patch (Text S1 for a description of these response variables).

## Supporting Information

Text S1 (DOCX)

Fig. S1 Distribution of remaining forest patches across the northern part of the study region in the Atlantic Forest of northeastern Brazil (inset shows the entire study region). Surveyed patches are shown in red (numbers refer to information listed in Table S1).
(TIFF)

Fig. S2 Distribution of remaining forest patches across the central part of study region in the Atlantic Forest of northeastern Brazil (inset shows the entire study region). Surveyed patches are shown in red (numbers refer to information listed in Table S1).
(TIFF)

Fig. S3 Distribution of remaining forest patches across the s part of study region in the Atlantic Forest of northeastern Brazil (inset shows the entire study region). Surveyed patches are shown in red (numbers refer to information listed in Table S1).
(TIFF)

Table S1 Geographic location and total area of each of 196 forest patches surveyed across the entire study region of the Brazilian Atlantic Forest. **Code**: Forest patches are numbered according to the maps available in Appendix III. **Map**: Alphanumeric code for Figure 1 (main text) and maps in Appendix III. **S**: total number of mammal species. Sites within municipal counties, states and biogeographic subregions (BSRs) marked with an asterisk (*) had been strictly protected for at least 8 years.
(DOCX)

Table S2 Mean, minimum and maximum area (ha) for six size classes of forest patches sampled, and the mean percentage of mammal species (*N* = 18) known to occupy those patches.
(DOCX)

Table S3 AICc-based model selection based on a candidate set of models predicting large mammal species richness, aggregate mammal biomass, and a composite metric of vulnerability of species retained in 196 forest patches of the Atlantic Forest of northeastern Brazil. For each model the number of parameters (K), AIC score corrected for small sample size (AIC*_c_*), AIC*_c_* difference from the best model (Δ AIC*_c_*) and Akaike weight (w*i*) are shown.
(DOCX)

Table S4 Averaged coefficient estimates, unconditional standard errors, unconditional variance, and relative importance of averaged coefficients calculated for aggregate properties of mammal assemblages retained within 196 forest patches of the Brazilian Atlantic forest over all models retained in the final candidate set.
(DOCX)
