# Supplementary material for: Pervasive Defaunation of Forest Remnants in a Tropical Biodiversity Hotspot
Source: PLoS One. 2012 Aug 14;7(8):e41671. doi: 10.1371/journal.pone.0041671 (PMC3419225; doi:10.1371/journal.pone.0041671)
Supplement: Text S1 — (DOCX) [file pone.0041671.s001.docx]

**Text S1**

**Supporting Information**

**Pervasive defaunation of forest remnants in a tropical biodiversity hotspot**

G.R. Canale, C.A. Peres, C.E. Guidorizzi, C.A.F. Gatto and M.C.M. Kierulff

**Study area and forest patch selection**

Between January 2003 and January 2005, we used a 4WD-vehicle to travel over 205,000 km along paved and unpaved roads within a 252,669-km^2^ subregion of the Atlantic Forest of northeastern Brazil, between the southern bank of the São Francisco River and the northern bank of the Jequitinhonha River, covering large parts of three Brazilian states, including Bahia, Sergipe and eastern Minas Gerais (Fig.1, Fig. S1-S3). In this vast study region, deforested areas dominated by cattle-ranch largeholdings were mainly concentrated farther inland from the coast, whereas smallholdings containing shaded cocoa (*Theobroma cacao*) agroforestry, conventional cocoa plantations, rubber tree (*Hevea brasiliensis*) plantations, and subsistence agriculture were the dominant land-uses along more coastal areas.

We used 23 satellite image (Landsat 7 ETM, 2001, UTM, Datum Córrego Alegre) with a 30-m resolution to both pre-select forest fragments to be surveyed and subsequently locate them during field campaigns using hardcopy maps and a portable laptop containing georeferenced images linked to a GPS (Garmin 60csx). Our exhaustive search across this vast region of both satellite images and maps, and forest remnants *in situ* led us to pinpoint and subsequently visit all large (>1,000 ha), most medium, and many small forest fragments, which numerically dominated the study region (see main text). Using a GPS, we were able to plot our locations *in situ* in relation to each target forest patch that we selected. We were also able to map all households within the immediate neighborhood of each patch, which were approached if they contained potential local informants with long-term knowledge of the history and mammal species occupancy of each forest patch.

Our sampling strategy ensured that forest fragments visited were evenly spread across the entire study region (see main text). Only five forest patches surveyed consisted of formal strictly protected areas (Biological Reserves and analogous parks) that had been relatively well-established and managed by government agencies. These were created by federal agencies between 1980 and 2003 under different forest reserve denominations (Table S1), whereby *de facto* law enforcement with regular boundary patrols since reserve creation had effectively inhibited hunting, logging, other timber and non-timber extractive activities, as well as agricultural encroachment. All other forest patches surveyed, although in reasonably good condition in terms of forest canopy structure and profile, were either forest set-asides within private landholdings that had been partially protected to a highly variable degree, or multifunctional ‘soft’ conservation areas effectively lacking law enforcement (e.g. APAs, *Environmental Protection Areas*). These forest patches therefore remained vulnerable to a wide range of direct and indirect human impacts and are defined as ‘unprotected’ in this study, despite the fact that their forest cover *per se* had been largely protected*.*

**Patch-scale species occupancy**

During site visits, we conducted 408 local interviews to obtain mammal species occupancy (presence/absence) data within a total of 196 variable-sized forest patches (size range = 0.17 – 194,341 ha). Following a field-tested protocol [1], these data are based on semi-structured interviews with long-term local residents (mostly adult men aged 51.8 ± 9.8 yrs-old) who had lived in the immediate vicinities of any given focal forest patch, and were completely familiar with both the large vertebrate fauna and the contemporary disturbance history of a given neighboring patch for at least 5 years (mean local residence time = 47.6 ± 10.3 yrs). We selected 196 interviews (one for each patch) from a total of 408 informants interviewed using the same protocol. We selected the ‘best’ informant to complete any given interview on the basis of four basic preconditions: 1) Informants had lived for more than 10 years near (often immediately adjacent to) a given forest fragment; 2) Informants were fully familiar with the local midsized to large mammal fauna; 3) Informants by necessity or otherwise had frequently entered the target forest fragment (at least once biweekly) during at least 2 years prior to interviews; and 4) Informants were willing to provide information following an informal approach by a member of our field team.

In total, this study synthesizes local information from a combined pool of 8,846 interviewee-years of continuous peri-patch residence time. To aid identification of large and midsized mammal species occurring in the surveyed patches, interviewees were shown during interviews clear illustrations, including the best available color plates and photos, of all terrestrial and arboreal mammal species (>1 kg) known to occur throughout this Atlantic Forest region. We selected mammal species larger than 1 kg because they were clearly most familiar to the local informants who had been approached during a set of pilot interviews conducted prior to the study. Although marmosets (*Callithrix* spp.) are smaller than 1 kg, we also included these small-bodied diurnal primates because they are conspicuous, widely familiar to local residents, and noteworthy in terms of regional scale conservation interest. The vast majority of local informants approached during this study had not traveled outside our study region, and were therefore unfamiliar with other mammal faunas. Because of possible omission or commission errors, we attempted to carefully frame questions addressed to any given species, and quantify the prevalence of both type I and type II errors, whereby interviewees may have either failed to recognize certain species known to occur in the region (false absences) or report species known to be absent in the region (false presences). To quantify the prevalence of type II errors, all interviewees were shown (in a random order interspersed with questions addressing target species) color plates of five additional neotropical forest mammal species (two primates, one ungulate, one canid and one diurnal caviomorph rodent) occurring in Amazonia and Mesoamerica, but not in the Atlantic Forest biome. In 2,038 of 2,040 trials during 408 interviews, local interviewees never falsely reported the local occupancy of a vertebrate species known not to occur in our study region. Two questionable interviews that did contain one incidence each of type II errors were therefore excluded from the initial set of interviews. This indicates a high degree of reliability (100% in this case) in the occupancy data provided by the selected interviews for the 18 target species, or an overall propensity to commit type II (commission) errors that effectively equates to zero. We are therefore confident that all of our interviewees failed to affirm the occupancy of any species that they did not know about and, conversely, that the occupancies that they did report relate to species that they did know about locally.

We further verified our interview-based sampling protocol by conducting rapid (3 days) orthodox field surveys (including line-transect censuses, camera-trapping; sand track-plates; audio playbacks; and counts of armadillo burrows) of the midsized to large mammal fauna across 46 of the 196 fragments that we visited. In addition, a comprehensive line-transect survey effort was conducted at three large forest sites (Una Biological Reserve, Serra do Conduru State Park and Michelin Ecological Reserve; see Table S1), based on a 6-year study that surveyed those sites continuously on a weekly basis, 3-4 days per week. These intensive field surveys provide a 100% match to the species occupancy data (occurrences or local extinctions) reported by our interviewees. Play-backs during rapid surveys targeted only two vocally conspicuous primate genera (*Callithrix* spp. and *Callicebus* spp.), and confirmed species occurrences for these taxa in 89% and 72% of the sites for which they had been reported by local informants. In general, however, rapid surveys yielded a relatively species-poor mammal assemblage composition for all forest patches surveyed; of the 18 mammal species considered here, only 12 species reported by interviewees across all sites were actually unambiguously detected during diurnal line-transect censuses (*Callithrix kuhlii*, *Leontopithecus chrysomelas*, *Callicebus melanochir*, *Cebus xanthosternos, Eira Barbara, Nasua nasua, Bradypus torquatus*, *Dasypus* sp., *Tamandua tetradactyla, Pecari tajacu*, *Mazama* sp.*, Dasyprocta* sp.). Seven of these species were also camera-trapped (*Callithrix* *kuhlii*, *Leontopithecus* *chrysomelas*, *Cebus* *xanthosternos*, *Eira* *Barbara*, *Nasua* *nasua*, *Puma* *concolor*, *Pecari* *tajacu*). Species-specific counts of armadillo burrows averaged at an extremely low rate of only 0.3 burrows per fragment (for a subset of 40 fragments surveyed using this method), and only three species were recorded using sand track-plates (*Cerdocyon* *thous* at two sites; *Mazama* sp. at a single site; and *Pecari* *tajacu* at two sites). These low mammal encounter rates were also widely reported by local residents, who reported detections of very few sightings, vocalizations and animal tracks. In any case, all species sighted or otherwise recorded using any means of direct (visual and/or acoustic) detections or indirect evidence (e.g. tracks, burrows) had also been reported by local interviewees. Field surveys therefore contributed no additional species occupancy data to those obtained during interviews corresponding to the same forest fragments, which suggests a low overall rate of type I (omission) errors in the interview data.

Of the 22 vertebrate species occurring in the study region that we had initially assessed, we were able to obtain highly reliable data for 18 mammal species ranging across nearly three orders of magnitude in body mass (0.35 – 150 kg). We excluded four species of small and medium-sized cats (*Herpailurus yaguaroundi*, *Leopardus* *pardalis*, *L.* *wiedii* and *L.* *tigrinus*), because these occur at very low densities, are nocturnal or otherwise secretive, and often could not be reliably distinguished by our informants at the species level. Several focal ‘species’ groupings included in this study may in fact represent two closely-related congeners occurring either sympatrically (e.g. brocket deer, *Mazama* spp.) or as parapatric congeners separated by a hybrid zone or sharp range boundary within the study region (e.g. marmosets, *Callithrix* spp.). However, these congeners are treated here as functional groups or ‘eco-species’ because they (i) could not always be reliably differentiated at the species or subspecies level by informants, and (ii) effectively exhibited the same population-level ecological responses to forest habitat loss and isolation, and human disturbance.

Assessment of the original regional scale geographic distribution of the study species were based on IUCN (2009) maps, interviews with local people, and information from mammalogists who have worked throughout the study region. There is little uncertainty about the extent of the geographic range distribution of the species considered here, with the exception of woolly spider monkeys (*Brachyteles hypoxanthus*). Although some primatologists believe that the Jeriquiça river is the northern range limit for this species [2, 3], this is not entirely clear especially considering that there are no geographic or ecological barriers to the west of our study region, at least until the Chapada Diamantina montane range. Moreover, the longitudinal range boundary for this species in the southern part of its distribution is even farther west of the headwaters of Jequiriça river basin. This large-bodied primate had no obvious ecological barrier within our study region, and it likely occurred in both continuous evergreen forests and mesic gallery forests within seasonally-dry forests of the entire northern Atlantic Forest domain during pre-Columbian times.

**Data Analysis.** We assess the importance of patch- and landscape-scale environmental predictors on three response variables describing assemblage-wide properties of mammal species persisting in each patch: (1) the total species richness (*S*); (2) the aggregate body mass (Σ BMi) of all species persisting in any given patch; and (3) a measure of the aggregate species vulnerability to depletion by game hunters of these species [Σ (HPi * 1/λi )], where HP is a rank of hunter preference of different prey species across the study region, which was calculated from information obtained from all our interviews, and 1/λi is a measure of species fecundity, defined as the inverse of the species-specific intrinsic rate of population increase (λ). This measure therefore takes into account both the degree to which different species are pursued by game hunters, and their intrinsic resiliency to exploitation (i.e. likelihood of recovering from any period of sustained hunting pressure).

We evaluate the effects of a wide spectrum of explanatory variables describing the history of forest disturbance within fragments, the protection status conferred to each fragment, and the human population (household) density both within and around fragments (Table S4). Using Fragstats^®^, we calculated the patch size, patch shape and several landscape metrics associated with each forest isolate sampled. However, most of these metrics were highly inter-correlated (r > 0.75) and were therefore excluded from the models. Only three patch and landscape metrics summarizing the patch size and landscape context were selected (Table S4): patch area (ha), the mean proximity between patches (Prox index), and the total amount of primary and secondary forest remaining within a non-circular 1-km buffer zone outside the perimeter of each forets patch.

We initially evaluated the degree of spatial autocorrelation of all sites visited. The number and species identity of mammal assemblages (defined as the Bray-Curtis dissimilarity distance in species composition) retained at different patches were unrelated to the geographic location of all other patches (Mantel tests with 999 unconditional permutations, Global Pearson's r > 0.01 in both cases). We therefore assumed that our widely scattered forest patches (pairwise distances = 288,452 ± 158,871 m, *N* = 38,220 comparisons) could be treated as spatially independent. We then used generalized linear models to relate variation in response variables to environmental predictors. We calculated the log likelihood value, number of model parameters (K), AIC*_c_* values, ΔAIC*_c_*, and model probabilities following Burnham and Anderson [4]. We used a model selection approach to identify a subset of top models with strong levels of empirical support (ΔAIC_c_ < 2). We used AIC_c_ (AIC corrected for small sample sizes) in all analyses rather than AIC, as the number of forest patches we surveyed divided by the number of model parameters was <40 in all cases. We accounted for model uncertainty in multi-model inference by performing model averaging (*sensu* Ref. 8). The Akaike weight was used to rank the importance of variables and produce model-averaged parameter estimates [4]. The relative importance of each predictor can be evaluated using the sum of Akaike weights of each candidate model in which each predictor appears, resulting in a selection probability (Σ ***w_i_***) that a given predictor appears in the best approximating model [5]. Species richness was evaluated using a Poisson error structure after the degree of overdispersion had been assessed, and aggregate biomass and aggregate vulnerability were log-transformed (log_10_ (x + 1)) to satisfy model assumptions of normality, and then treated as a Gaussian process. We built models using R (version 2.9.1 10), and used the package *glmulti* to facilitate multi-model inference based on every possible first-order combination of predictor variables [6]. This package also calculates selection probabilities for each variable, from which their relative importance could be inferred. However, poor predictors are not expected to have selection probabilities close to zero [5]. We therefore incorporated a null predictor into the model, with which to gauge the relative explanatory power of predictors and identify those with a genuine effect on mammal persistence within forest patches [7].

We also constructed nonlinear species-area relationships (SARs) based on eight possible models using the R-package ‘mmSAR’ [8], including four convex (power, exponential, negative exponential, and Monod) and four sigmoidal models (rational function, logistic, Lomolino, and cumulative Weibull). We then used information theoretic analyses to evaluate the performance of the most parsimonious models using Akaike weights [4]. We initially avoided linearized forms of the SAR using log-transformations of the power and exponential models, as often used in the SAR literature [9]. These, however, were later linearized for the sake of illustration, without major loss of explanatory power.

**References**

1 Michalski F, Peres CA (2005) Anthropogenic determinants of primate and carnivore local extinctions in a fragmented forest landscape of southern Amazonia. Biol Conserv 124:383–396.

2 Aguirre AC (1971) O mono *Brachyteles arachnoides* (E. Geoffroy): A Situação Atual da Espécie no Brasil. Rio de Janeiro: Academia Brasileira de Ciências, Brazil. 53pp.

3 Mendes SL, Melo FR , Boubli JP, Dias LG, Strier KB et al. (2005) Directives for the conservation of the northern muriqui, *Brachyteles hypoxanthus* (Primates, Atelidae). Neotropical Primates 13: 7-18.

4 Burnham KP, Anderson DR (2002) Model selection and multimodel inference: a practical information-theoretic approach. New York: Springer. 488pp.

5 Whittingham MJ, Swetnam RD, Wilson JD, Chamberlain DE, Freckleton RP (2005) Habitat selection by yellowhammers (*Emberiza citrinella*) on lowland farmland at two spatial scales: Implications for conservation management, J. Appl. Ecol. **42:**270–280.

6 Calcagno V (2010) glmulti: GLM model selection and multimodel inference made easy. R package version 0.6-3. <http://CRAN.R-project.org/package=glmulti>.

7 Gray TNE, Borey R, Hout SK, Chamnan H, Collar NJ et al. (2009) Generality of Models that Predict the Distribution of Species: Conservation Activity and Reduction of Model Transferability for a Threatened Bustard. Conserv Biol 23:433-439.

8 Guilhaumon F, Mouillot D, Gimenez O (2010) mmSAR: an R-package for multimodel species-area relationship inference. Ecography 33:420-424.

9 Rosenzweig ML (1995) Species diversity in space and time. Cambridge: University Cambridge Press, Cambridge. 436pp.
