# Supplementary figures and images for: Pervasive Defaunation of Forest Remnants in a Tropical Biodiversity Hotspot
Source: PLoS One. 2012 Aug 14;7(8):e41671. doi: 10.1371/journal.pone.0041671 (PMC3419225; doi:10.1371/journal.pone.0041671)

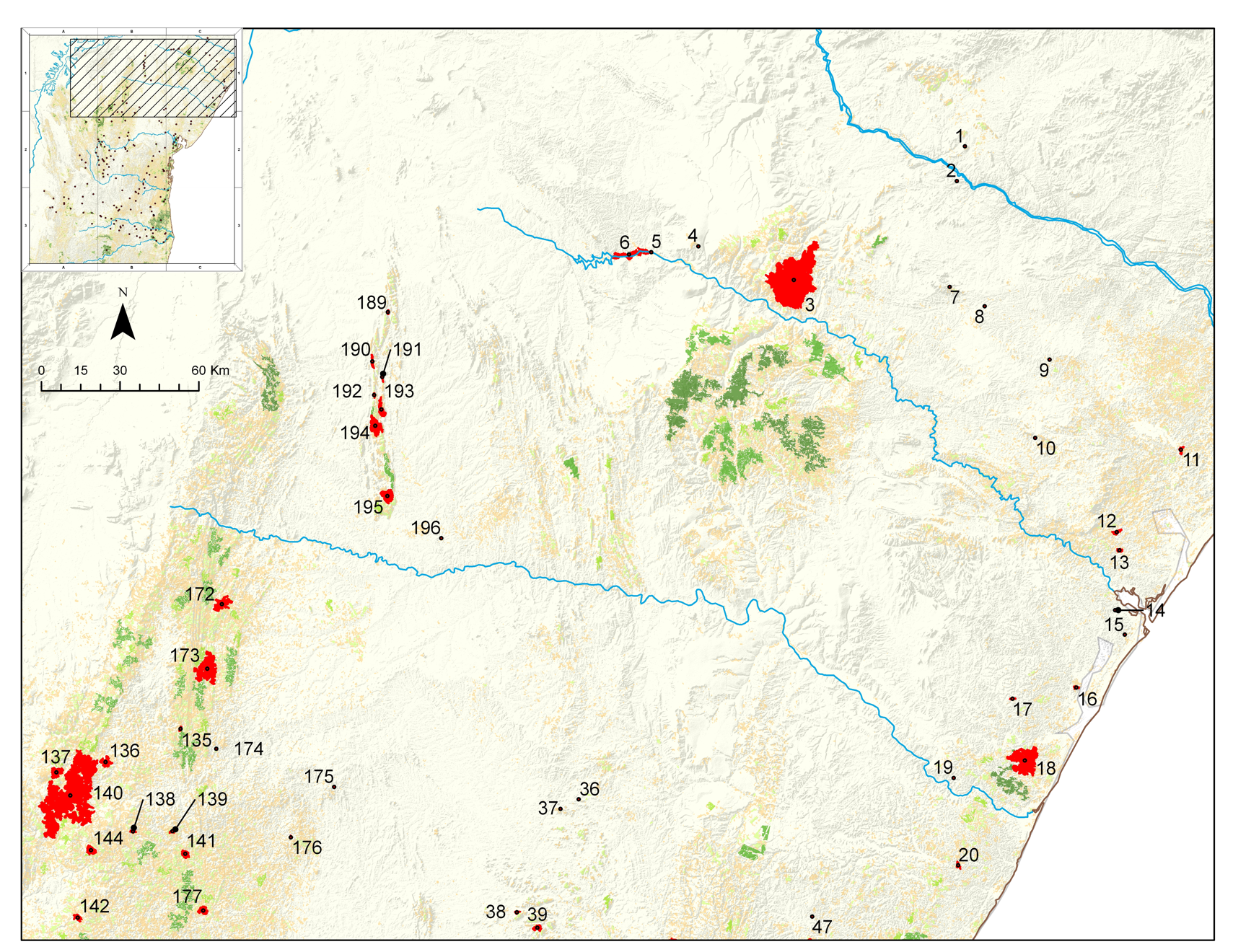

Supplement: Fig. S1 — Distribution of remaining forest patches across the northern part of the study region in the Atlantic Forest of northeastern Brazil (inset shows the entire study region). Surveyed patches are shown in red (numbers refer to information listed in Table S1). (TIFF) [file pone.0041671.s002.tiff]

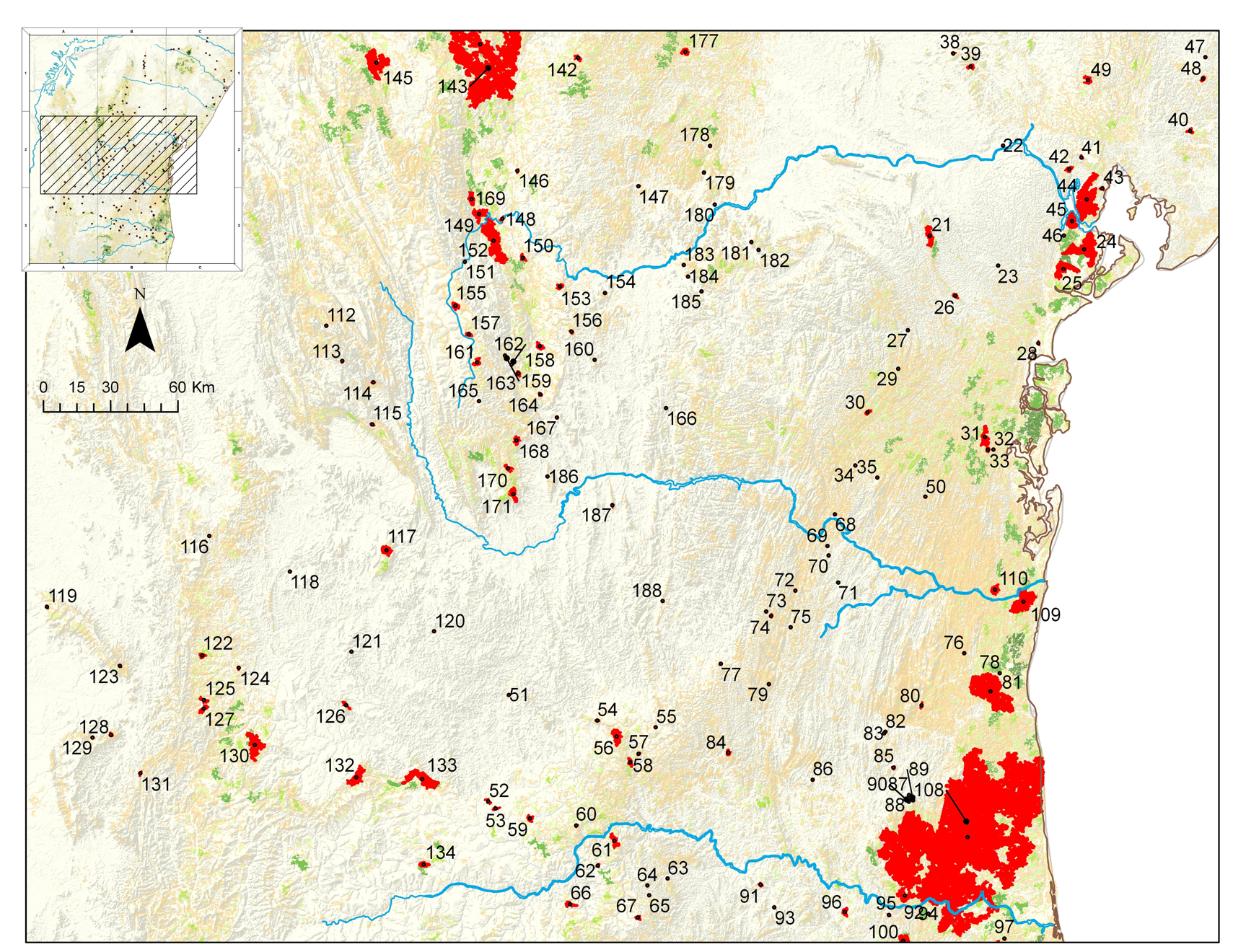

Supplement: Fig. S2 — Distribution of remaining forest patches across the central part of study region in the Atlantic Forest of northeastern Brazil (inset shows the entire study region). Surveyed patches are shown in red (numbers refer to information listed in Table S1). (TIFF) [file pone.0041671.s003.tiff]

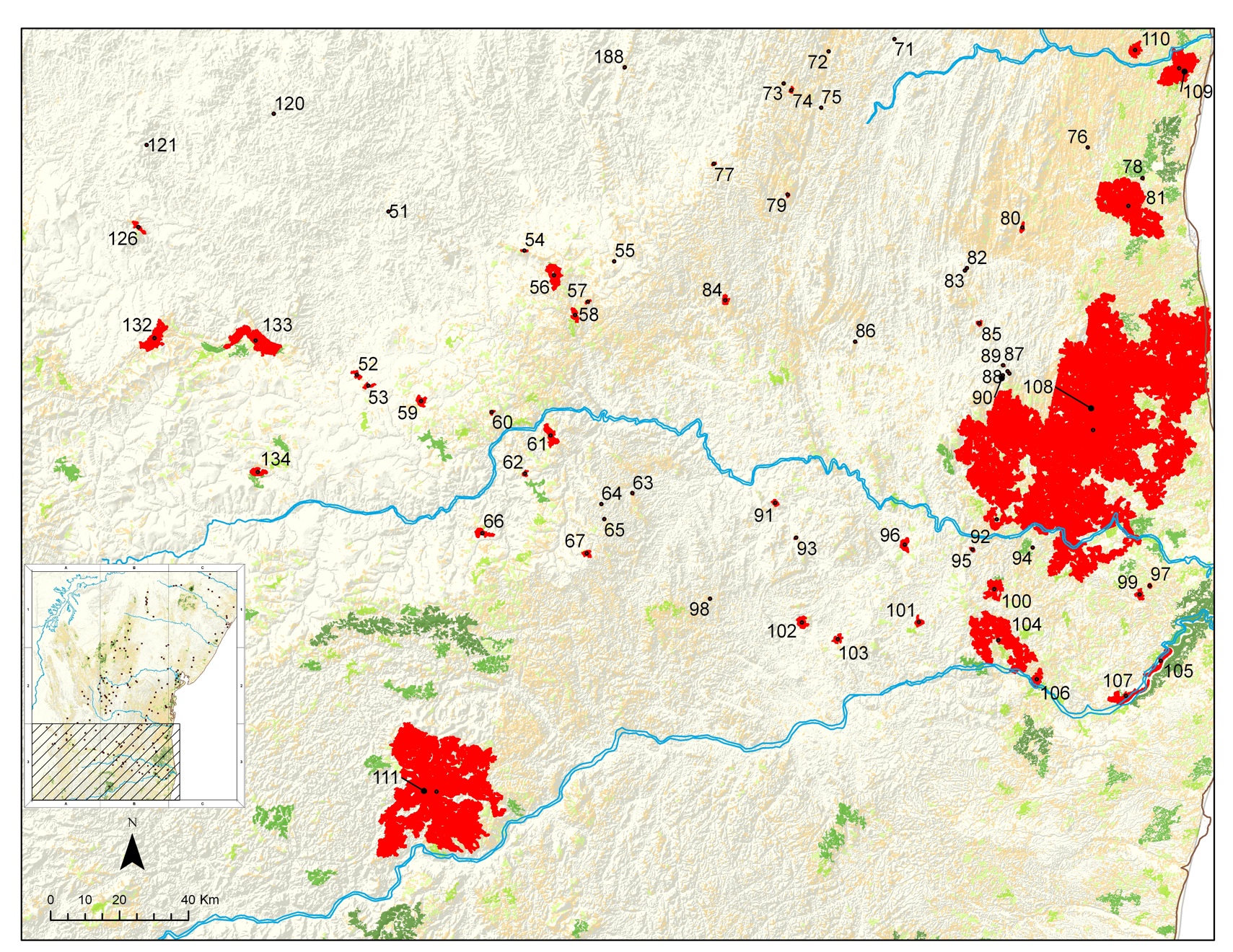

Supplement: Fig. S3 — Distribution of remaining forest patches across the s part of study region in the Atlantic Forest of northeastern Brazil (inset shows the entire study region). Surveyed patches are shown in red (numbers refer to information listed in Table S1). (TIFF) [file pone.0041671.s004.tiff]
