# Supplementary material for: Pervasive Defaunation of Forest Remnants in a Tropical Biodiversity Hotspot
Source: PLoS One. 2012 Aug 14;7(8):e41671. doi: 10.1371/journal.pone.0041671 (PMC3419225; doi:10.1371/journal.pone.0041671)
Supplement: Table S1 — Geographic location and total area of each of 196 forest patches surveyed across the entire study region of the Brazilian Atlantic Forest. Code: Forest patches are numbered according to the maps available in Appendix III. Map: Alphanumeric code for Figure 1 (main text) and maps in Appendix III. S: total number of mammal species. Sites within municipal counties, states and biogeographic subregions (BSRs) marked with an asterisk (*) had been strictly protected for at least 8 years. (DOCX) [file pone.0041671.s005.docx]

**Table S1** Geographic location and total area of each of 196 forest patches surveyed across the entire study region of the Brazilian Atlantic Forest. **Code**: Forest patches are numbered according to the maps available in Appendix III. **Map**: Alphanumeric code for Figure 1 (main text) and maps in Appendix III. **S**: total number of mammal species. Sites within municipal counties, states and biogeographic subregions (BSRs) marked with an asterisk (*) had been strictly protected for at least 8 years.

| **Code** | **Map** | **AREA (ha)** | **S** | **UTM (X)** | **UTM (Y)** | **Municipality** | **State** | **BSR** |
| --- | --- | --- | --- | --- | --- | --- | --- | --- |
| 1 | C1 | 0.8 | 2 | 628470 | 8950046 | Olhos d'água do Carado | Alagoas | Pernambuco |
| 2 | C1 | 0.2 | 1 | 626634 | 8937993 | Canindé de Sao Francisco | Sergipe | Bahia |
| 3 | C1 | 20,955.5 | 3 | 566597 | 8891315 | Jeremoabo | Bahia | Bahia |
| 4 | C1 | 20.9 | 1 | 566597 | 8891315 | Jeremoabo | Bahia | Bahia |
| 5 | C1 | 20.9 | 0 | 496000 | 8908000 | Canudos | Bahia | Bahia |
| 6 | B1/C1 | 1,197.5 | 2 | 528000 | 8912000 | Canudos | Bahia | Bahia |
| 7 | C1 | 6.2 | 1 | 624107 | 8898475 | Poço Redondo | Sergipe | Bahia |
| 8 | C1 | 0.3 | 5 | 637682 | 8889093 | Monte Alegre | Sergipe | Bahia |
| 9 | C1 | 12.8 | 2 | 663000 | 8870900 | Nossa Senhora da Glória | Sergipe | Bahia |
| 10 | C1 | 2.4 | 4 | 656772 | 8838651 | Frei Paulo | Sergipe | Bahia |
| 11 | C1 | 176.2 | 1 | 712229 | 8834398 | Capela | Sergipe | Bahia |
| 12 | C1 | 206.9 | 3 | 598075 | 8812489 | Poço Verde | Sergipe | Bahia |
| 13 | C1 | 125.1 | 2 | 687911 | 8797399 | Areia Branca | Sergipe | Bahia |
| 14 | C1 | 1.0 | 6 | 689353 | 8772087 | Itaporanga | Sergipe | Bahia |
| 15 | C1 | 32.0 | 6 | 690611 | 8764216 | Itaporanga | Sergipe | Bahia |
| 16 | C1 | 124.0 | 4 | 673097 | 8739675 | Santa Luzia do Itanhy | Sergipe | Bahia |
| 17 | C1 | 52.2 | 2 | 646559 | 8741352 | Umbaúba | Sergipe | Bahia |
| 18 | C1 | 5,581.1 | 6 | 658358 | 8720187 | Jandaíra | Bahia | Bahia |
| 19 | C1 | 9.3 | 4 | 625887 | 8708879 | Esplanada | Bahia | Bahia |
| 20 | C2 | 132.0 | 6 | 628203 | 8675010 | Esplanada | Bahia | Bahia |
| 21 | B2 | 1,072.7 | 3 | 447725 | 8576980 | Elísio Medrado | Bahia | Bahia |
| 22 | B2/C2 | 1.8 | 3 | 479536 | 8615648 | Cabeceiras do Paraguaçu | Bahia | Bahia |
| 23 | B2/C2 | 20.8 | 3 | 480158 | 8562596 | Santo Antônio de Jesus | Bahia | Bahia |
| 24 | C2 | 6,281.2 | 3 | 511708 | 8571838 | Nazaré das Farinhas | Bahia | Bahia |
| 25 | C2 | 2,580.3 | 3 | 509590 | 8564399 | Nazaré das Farinhas | Bahia | Bahia |
| 26 | B2/C2 | 227.0 | 4 | 447725 | 8576980 | Elísio Medrado | Bahia | Bahia |
| 27 | B2 | 12.0 | 3 | 437358 | 8534317 | Jequiriçá | Bahia | Bahia |
| 28 | B2/C2 | 48.7 | 3 | 498610 | 8527088 | Guaibim/Valença | Bahia | Bahia |
| 29 | B2 | 16.1 | 6 | 461281 | 8547690 | Lage | Bahia | Bahia |
| 30 | B2 | 197.6 | 10 | 422099 | 8496914 | Wenceslau Guimaraes* | Bahia | Bahia |
| 31 | B2/C2 | 1,131.1 | 4 | 474153 | 8485806 | Nilo Peçanha | Bahia | Bahia |
| 32 | B2/C2 | 18.2 | 3 | 476855 | 8480614 | Ituberá | Bahia | Bahia |
| 33 | B2/C2 | 163.9 | 6 | 475445 | 8479059 | Ituberá | Bahia | Bahia |
| 34 | B2 | 36.7 | 3 | 415727 | 8473168 | Apuarema | Bahia | Bahia |
| 35 | B2 | 1.7 | 5 | 425889 | 8468219 | Itamari | Bahia | Bahia |
| 36 | B1/C1 | 20.3 | 2 | 483962 | 8700619 | Ichu | Bahia | Diamantina |
| 37 | B1/C1 | 18.5 | 2 | 475740 | 8697624 | Ichu | Bahia | Diamantina |
| 38 | B1/B2/C2 | 103.6 | 3 | 462076 | 8658913 | Serra Preta | Bahia | Diamantina |
| 39 | B1/B2/C2 | 267.0 | 5 | 465571 | 8652100 | Serra Preta | Bahia | Diamantina |
| 40 | C2 | 259.4 | 6 | 565672 | 8625148 | Pojuca/ Catu | Bahia | Bahia |
| 41 | C2 | 60.6 | 3 | 518738 | 8611464 | Santo Amaro | Bahia | Bahia |
| 42 | C2 | 327.8 | 4 | 511920 | 8608194 | Cachoeira | Bahia | Bahia |
| 43 | C2 | 155.7 | 6 | 526783 | 8598438 | Itapema | Bahia | Bahia |
| 44 | C2 | 7,976.0 | 6 | 514181 | 8591242 | Cachoeira | Bahia | Bahia |
| 45 | C2 | 1,827.2 | 5 | 511748 | 8579859 | Sao Roque do Paraguaçu | Bahia | Bahia |
| 46 | C2 | 25.4 | 3 | 508302 | 8577259 | Guaí | Bahia | Bahia |
| 47 | C2 | 2.4 | 2 | 569511 | 8654986 | Alagoinha / Araças | Bahia | Bahia |
| 48 | C2 | 203.4 | 4 | 568775 | 8645053 | Catu | Bahia | Bahia |
| 49 | C2 | 568.5 | 2 | 519029 | 8648299 | Coraçao de Maria | Bahia | Bahia |
| 50 | B2 | 12.2 | 8 | 447737 | 8460000 | Gandu | Bahia | Bahia |
| 51 | A3/B3 | 0.8 | 3 | 256755 | 8370481 | Caraíbas | Bahia | Diamantina |
| 52 | A3/B3 | 256.9 | 2 | 247692 | 8321681 | Quaraçu | Bahia | Diamantina |
| 53 | A3/B3 | 193.9 | 3 | 257978 | 8317136 | Lagoa da Timóteo | Bahia | Diamantina |
| 54 | B3 | 59.0 | 2 | 302652 | 8359445 | Vitória da Conquista | Bahia | Diamantina |
| 55 | B3 | 16.4 | 7 | 330109 | 8356620 | Barra do Choça | Bahia | Diamantina |
| 56 | C3 | 1,425.9 | 3 | 313754 | 8350010 | Vitória da Conquista | Bahia | Diamantina |
| 57 | B3 | 45.7 | 3 | 320193 | 8345845 | Vitória da Conquista | Bahia | Diamantina |
| 58 | B3 | 305.9 | 3 | 318832 | 8340420 | Barra do Choça | Bahia | Diamantina |
| 59 | A3/B3 | 460.5 | 3 | 274294 | 8314801 | Vitória da Conquista | Bahia | Diamantina |
| 60 | B3 | 71.5 | 4 | 291296 | 8312164 | Inhobim | Bahia | Diamantina |
| 61 | B3 | 937.4 | 8 | 316715 | 8305631 | Itambé | Bahia | Diamantina |
| 62 | B3 | 116.3 | 7 | 300772 | 8296608 | Ribeirão do Largo | Bahia | Diamantina |
| 63 | B3 | 24.8 | 1 | 328704 | 8289552 | Nova Brasília | Bahia | Diamantina |
| 64 | B3 | 3.1 | 3 | 323657 | 8286733 | Nova Brasília | Bahia | Diamantina |
| 65 | B3 | 17.7 | 1 | 322639 | 8282216 | Nova Brasília | Bahia | Diamantina |
| 66 | B3 | 596.6 | 7 | 295462 | 8281780 | Encruzilhada | Bahia | Diamantina |
| 67 | B3 | 258.8 | 6 | 316765 | 8271521 | Vila do Café | Bahia | Diamantina |
| 68 | B2 | 31.6 | 2 | 407397 | 8450988 | Jitaúna | Bahia | Bahia |
| 69 | B2 | 2.9 | 7 | 404077 | 8437486 | Aiquara | Bahia | Bahia |
| 70 | B2/B3 | 20.4 | 1 | 404077 | 8432486 | Aiquara | Bahia | Bahia |
| 71 | B3 | 0.8 | 4 | 408777 | 8420329 | Itajubá | Bahia | Bahia |
| 72 | B3 | 6.5 | 2 | 389781 | 8417696 | Ponto Novo | Bahia | Bahia |
| 73 | B3 | 13.0 | 3 | 375935 | 8407431 | Boa Nova | Bahia | Bahia |
| 74 | B3 | 53.4 | 7 | 378900 | 8405310 | Boa Nova | Bahia | Bahia |
| 75 | B3 | 5.5 | 3 | 387026 | 8402137 | Boa nova | Bahia | Bahia |
| 76 | B3 | 1.5 | 1 | 464500 | 8390000 | Uruçuca | Bahia | Bahia |
| 77 | B3 | 56.3 | 4 | 352774 | 8393333 | Poções | Bahia | Diamantina |
| 78 | B3 | 43.4 | 2 | 480000 | 8380000 | Ilhéus | Bahia | Bahia |
| 79 | B3 | 41.1 | 8 | 383000 | 8368000 | Iguaí | Bahia | Diamantina |
| 80 | B3 | 118.4 | 0 | 445210 | 8365231 | Lomanto Júnior | Bahia | Bahia |
| 81 | B3 | 10,947.7 | 3 | 472050 | 8377100 | Ilhéus | Bahia | Bahia |
| 82 | B3 | 0.4 | 1 | 428726 | 8354127 | Floresta Azul | Bahia | Bahia |
| 83 | B3 | 0.3 | 2 | 428726 | 8354127 | Floresta Azul | Bahia | Bahia |
| 84 | B3 | 201.5 | 3 | 348900 | 8343800 | Caatiba | Bahia | Diamantina |
| 85 | B3 | 82.0 | 3 | 435110 | 8338573 | Jussari | Bahia | Bahia |
| 86 | B3 | 29.6 | 2 | 402000 | 8310513 | Itajú | Bahia | Bahia |
| 87 | B3 | 26.1 | 2 | 439500 | 8326600 | Palmira | Bahia | Bahia |
| 88 | B3 | 9.3 | 1 | 441210 | 8324810 | Juçari | Bahia | Bahia |
| 89 | B3 | 2.3 | 1 | 442011 | 8324121 | Juçari | Bahia | Bahia |
| 90 | B3 | 0.6 | 1 | 440000 | 8324000 | Palmira | Bahia | Bahia |
| 91 | B3 | 149.5 | 3 | 374854 | 8284017 | Itarantim | Bahia | Diamantina |
| 92 | B3 | 1,292.3 | 5 | 406716 | 8275487 | Potiraguá | Bahia | Bahia |
| 93 | B3 | 37.3 | 6 | 378415 | 8276790 | Itarantim | Bahia | Diamantina |
| 94 | B3 | 1.0 | 6 | 450264 | 8416248 | Gongogi | Bahia | Bahia |
| 95 | B3 | 79.5 | 1 | 433268 | 8272859 | Potiraguá | Bahia | Bahia |
| 96 | B3 | 528.4 | 3 | 410141 | 8269141 | Potiraguá | Bahia | Bahia |
| 97 | B3 | 71.8 | 2 | 482925 | 8262469 | Canavieiras | Bahia | Bahia |
| 98 | B3 | 0.2 | 2 | 457006 | 8408715 | Banco Central | Bahia | Diamantina |
| 99 | B3 | 309.9 | 1 | 478003 | 8258231 | Canavieiras | Bahia | Bahia |
| 100 | B3 | 1,241.2 | 3 | 439678 | 8263030 | Potiraguá | Bahia | Bahia |
| 101 | B3 | 342.0 | 2 | 414906 | 8255381 | Potiraguá | Bahia | Diamantina |
| 102 | B3 | 584.0 | 6 | 386264 | 8256477 | Itarantim | Bahia | Diamantina |
| 103 | B3 | 333.5 | 7 | 395189 | 8245525 | Itarantim | Bahia | Diamantina |
| 104 | B3 | 9,609.6 | 5 | 440812 | 8251067 | Itapebi | Bahia | Bahia |
| 105 | B3 | 918.8 | 2 | 486002 | 8245034 | Canavieiras | Bahia | Bahia |
| 106 | B3 | 650.3 | 3 | 444222 | 8233736 | Itapebi | Bahia | Bahia |
| 107 | B3 | 1,549.6 | 5 | 512655 | 8245986 | Belmonte | Bahia | Bahia |
| 108 | B3 | 194,341.3 | 12 | 486800 | 8321300 | Una* | Bahia | Bahia |
| 109 | B3 | 4,766.3 | 10 | 489838 | 8404102 | Serra Grande* | Bahia | Bahia |
| 110 | B3 | 654.8 | 11 | 479625 | 8419325 | Maraú | Bahia | Bahia |
| 111 | B3 | 63,441.9 | 16 | 178900 | 8228745 | Jequitinhonha* | Minas Gerais | Diamantina |
| 112 | A2 | 16.1 | 2 | 182000 | 8534000 | Piata | Bahia | Diamantina |
| 113 | A2 | 60.6 | 1 | 187000 | 8517000 | Rio de Contas | Bahia | Diamantina |
| 114 | A2 | 57.1 | 5 | 200000 | 8512000 | Rio de Contas | Bahia | Diamantina |
| 115 | A2 | 107.6 | 6 | 202000 | 8492000 | Marcolino Moura | Bahia | Diamantina |
| 116 | A2 | 19.3 | 2 | 775308 | 8441078 | Caetité | Bahia | São Francisco |
| 117 | A2/B3 | 1,109.9 | 5 | 207000 | 8432000 | Brumado | Bahia | Diamantina |
| 118 | A2 | 6.9 | 5 | 818644 | 8428611 | Rio do Antônio | Bahia | São Francisco |
| 119 | A3 | 52.5 | 3 | 700195 | 8415712 | Palmas de Monte Alto | Bahia | São Francisco |
| 120 | A3/B3 | 2.2 | 2 | 223957 | 8394781 | Maetinga | Bahia | São Francisco |
| 121 | A3 | 2.2 | 6 | 189023 | 8393697 | Guajerú | Bahia | São Francisco |
| 122 | A3 | 253.1 | 4 | 754579 | 8388163 | Urandi | Bahia | São Francisco |
| 123 | A3 | 80.1 | 8 | 733208 | 8393254 | Pindaí | Bahia | São Francisco |
| 124 | A3 | 59.3 | 4 | 802459 | 8389733 | Caculé | Bahia | São Francisco |
| 125 | A3 | 340.6 | 5 | 759692 | 8368047 | Urandi | Bahia | São Francisco |
| 126 | A3 | 243.9 | 3 | 209487 | 8362334 | Presidente Jânio Quadros | Bahia | São Francisco |
| 127 | A3 | 388.5 | 7 | 770594 | 8363466 | Vacaraci | Bahia | São Francisco |
| 128 | A3 | 124.0 | 6 | 734507 | 8355448 | Espinosa | Bahia | São Francisco |
| 129 | A3 | 3.8 | 6 | 723160 | 8357148 | Espinosa | Bahia | São Francisco |
| 130 | A3 | 3,198.2 | 5 | 794281 | 8341660 | Mortugaba | Bahia | São Francisco |
| 131 | A3 | 45.1 | 7 | 743937 | 8339007 | Espinosa | Bahia | São Francisco |
| 132 | A3 | 2,193.8 | 2 | 181168 | 8329894 | Cordeuros | Bahia | São Francisco |
| 133 | A3/B3 | 4,404.1 | 5 | 236871 | 8343933 | Tremedal | Bahia | São Francisco |
| 134 | A3/B3 | 635.0 | 2 | 214415 | 8300079 | Ninheiras | Bahia | Diamantina |
| 135 | B1 | 79.6 | 2 | 331679 | 8728953 | Miguel Calmon | Bahia | Bahia |
| 136 | B1 | 544.1 | 3 | 304544 | 8718321 | Morro do Chapéu | Bahia | Bahia |
| 137 | B1 | 691.1 | 3 | 282674 | 8708636 | Morro da Chápeu | Bahia | Bahia |
| 138 | B1 | 102.5 | 2 | 311399 | 8688499 | Tapiramutá | Bahia | Bahia |
| 139 | B1 | 109.1 | 6 | 330312 | 8692585 | Mundo Novo | Bahia | Bahia |
| 140 | B1 | 20,843.9 | 2 | 299413 | 8713515 | Morro do Chapéu | Bahia | Bahia |
| 141 | B1 | 374.3 | 7 | 334577 | 8679917 | Mundo Novo | Bahia | Bahia |
| 142 | B1/B2 | 243.8 | 4 | 290899 | 8655941 | Rui Barbosa | Bahia | Bahia |
| 143 | A2/B1/B2 | 52,109.4 | 7 | 234681 | 8665933 | Mulungú do Mono | Bahia | Bahia |
| 144 | B1 | 482.4 | 2 | 296989 | 8682721 | Tapiramutá | Bahia | Bahia |
| 145 | A2/B1 | 4,202.7 | 1 | 215383 | 8645147 | Iraquara | Bahia | Bahia |
| 146 | A2/B2 | 82.3 | 3 | 266109 | 8609510 | Andaraí | Bahia | Bahia |
| 147 | B2 | 32.6 | 2 | 320728 | 8599987 | Boa Vista do Tupim | Bahia | Bahia |
| 148 | A2/B2 | 245.2 | 2 | 258715 | 8585219 | Andaraí | Bahia | Bahia |
| 149 | A2/B2 | 1,355.5 | 7 | 240287 | 8610181 | Lençóis | Bahia | Bahia |
| 150 | A2/B2 | 270.4 | 2 | 271590 | 8568036 | Itueté | Bahia | Bahia |
| 151 | A2/B2 | 9.9 | 4 | 243000 | 8561000 | Mucugê | Bahia | Bahia |
| 152 | A2/B2 | 6,140.7 | 10 | 256043 | 8569567 | Mucugê | Bahia | Bahia |
| 153 | B2 | 330.6 | 1 | 288641 | 8557383 | Itaeté | Bahia | Bahia |
| 154 | B2 | 36.6 | 3 | 307371 | 8550628 | Marcionílio Sousa | Bahia | Bahia |
| 155 | A2/B2 | 410.3 | 4 | 240756 | 8548278 | Mucugê | Bahia | Bahia |
| 156 | B2 | 102.4 | 1 | 289857 | 8531729 | Iramaia | Bahia | Bahia |
| 157 | A2/B2 | 198.7 | 5 | 246000 | 8534000 | Ibicoara | Bahia | Bahia |
| 158 | A2/B2 | 506.5 | 8 | 277000 | 8526000 | Iramaia | Bahia | Bahia |
| 159 | A2/B2 | 30.2 | 1 | 260356 | 8520949 | Ibicoara | Bahia | Bahia |
| 160 | B2 | 28.7 | 6 | 295974 | 8521557 | Iramaia | Bahia | Bahia |
| 161 | A2/B2 | 390.4 | 4 | 246000 | 8523000 | Ibicoara | Bahia | Bahia |
| 162 | A2/B2 | 37.5 | 2 | 262686 | 8518634 | Livramento | Bahia | Bahia |
| 163 | A2/B2 | 271.6 | 2 | 262500 | 8512000 | Iramaia | Bahia | Bahia |
| 164 | A2/B2 | 119.3 | 3 | 277687 | 8504390 | Iramaia | Bahia | Bahia |
| 165 | A2/B2 | 12.4 | 4 | 248000 | 8503000 | Barra do Estiva | Bahia | Bahia |
| 166 | B2 | 21.9 | 4 | 329034 | 8503882 | Maracás | Bahia | Bahia |
| 167 | B2 | 4.5 | 7 | 285109 | 8498022 | Barra da Estiva | Bahia | Bahia |
| 168 | A2/B2 | 527.4 | 5 | 268618 | 8483987 | Conteuda-Ituaçu | Bahia | Bahia |
| 169 | A2/B2 | 889.0 | 13 | 244462 | 8592998 | Lençóis* | Bahia | Bahia |
| 170 | A2/B2 | 397.0 | 5 | 264000 | 8476000 | Contendas do Sincoró | Bahia | Bahia |
| 171 | A2/B2 | 790.9 | 4 | 267798 | 8459529 | Contendas do Sincorá | Bahia | Bahia |
| 172 | B1 | 1,110.1 | 5 | 344326 | 8773276 | Caém | Bahia | Bahia |
| 173 | B1 | 3,356.7 | 3 | 343896 | 8744834 | Jacobina | Bahia | Bahia |
| 174 | B1 | 12.8 | 2 | 344893 | 8720523 | Miguel Calmon | Bahia | Bahia |
| 175 | B1 | 0.3 | 2 | 390130 | 8706211 | Mairi | Bahia | Bahia |
| 176 | B1 | 43.9 | 6 | 373214 | 8687791 | Baixa Grande | Bahia | Bahia |
| 177 | B1/B2 | 464.3 | 4 | 339736 | 8659417 | Macajuba | Bahia | Bahia |
| 178 | B2 | 14.1 | 3 | 349791 | 8616153 | Itaberaba | Bahia | Bahia |
| 179 | B2 | 11.1 | 3 | 349774 | 8604377 | Itaberaba | Bahia | Bahia |
| 180 | B2 | 3.2 | 10 | 351997 | 8590187 | Boa Vista do Tupim | Bahia | Bahia |
| 181 | B2 | 37.1 | 3 | 368279 | 8572416 | Iaçú | Bahia | Bahia |
| 182 | B2 | 15.7 | 2 | 374076 | 8570401 | Iaçú | Bahia | Bahia |
| 183 | B2 | 1.9 | 1 | 339577 | 8562800 | Marcionílio Sousa | Bahia | Bahia |
| 184 | B2 | 1.3 | 6 | 339995 | 8557734 | Marcionílio Sousa | Bahia | Bahia |
| 185 | B2 | 5.4 | 5 | 347647 | 8551429 | Planaltino | Bahia | Bahia |
| 186 | B2 | 19.3 | 2 | 281000 | 8470000 | Contendas do Sincorá | Bahia | Bahia |
| 187 | B2 | 66.8 | 4 | 307000 | 8454000 | Manuel Vitorino | Bahia | Bahia |
| 188 | B3 | 29.1 | 4 | 324000 | 8414000 | Brumado | Bahia | Bahia |
| 189 | B1 | 79.3 | 5 | 410863 | 8888691 | Jaguarari | Bahia | Bahia |
| 190 | B1 | 218.4 | 10 | 403092 | 8869569 | Andorinha | Bahia | Bahia |
| 191 | B1 | 109.0 | 3 | 407153 | 8862222 | Andorinha | Bahia | Bahia |
| 192 | B1 | 48.4 | 2 | 405259 | 8853807 | Andorinha | Bahia | Bahia |
| 193 | B1 | 596.2 | 4 | 412741 | 8846703 | Andorinha | Bahia | Bahia |
| 194 | B1 | 1,390.2 | 3 | 409815 | 8841972 | Itiúba | Bahia | Bahia |
| 195 | B1 | 1,269.2 | 2 | 411746 | 8819253 | Itiúba | Bahia | Bahia |
| 196 | B1 | 0.8 | 4 | 430845 | 8801376 | Queimadas | Bahia | Bahia |
