# Supplementary material for: Pervasive Defaunation of Forest Remnants in a Tropical Biodiversity Hotspot
Source: PLoS One. 2012 Aug 14;7(8):e41671. doi: 10.1371/journal.pone.0041671 (PMC3419225; doi:10.1371/journal.pone.0041671)
Supplement: Table S2 — Mean, minimum and maximum area (ha) for six size classes of forest patches sampled, and the mean percentage of mammal species (N = 18) known to occupy those patches. (DOCX) [file pone.0041671.s006.docx]

**Table S2**. Mean, minimum and maximum area (ha) for six size classes of forest patches sampled, and the mean percentage of mammal species (*N* = 18) known to occupy those patches.

| **Patch area (ha)** | **Area (ha)** | | | **Species occupancy (%)** | **N** |
| --- | --- | --- | --- | --- | --- |
|  | **Mean ± SD** | **Minimum** | **Maximum** | **Mean ± SD** |  |
| 0-50 | 15.8 ± 14.5 | 0.2 | 48.7 | 18.2 ± 11.4 | 81 |
| 51-100 | 66.9 ± 11.3 | 52.2 | 82.3 | 19.4 ± 10.5 | 18 |
| 101-500 | 240.3 ± 108.1 | 102.4 | 482.4 | 22.2 ± 12.2 | 50 |
| 501-1000 | 663.7 ± 143.6 | 506.4 | 937.4 | 27.8 ± 18.4 | 16 |
| 1001-5000 | 2,083.7 ± 1,226.9 | 1,072.7 | 4,766.3 | 22.2 ± 11.5 | 20 |
| >5001 | 36,202.6 ± 55,990.7 | 5,581.1 | 194,341.3 | 39.9 ± 23.0 | 11 |
