# Supplementary material for: Pervasive Defaunation of Forest Remnants in a Tropical Biodiversity Hotspot
Source: PLoS One. 2012 Aug 14;7(8):e41671. doi: 10.1371/journal.pone.0041671 (PMC3419225; doi:10.1371/journal.pone.0041671)
Supplement: Table S3 — AICc-based model selection based on a candidate set of models predicting large mammal species richness, aggregate mammal biomass, and a composite metric of vulnerability of species retained in 196 forest patches of the Atlantic Forest of northeastern Brazil. For each model the number of parameters (K), AIC score corrected for small sample size (AICc), AICc difference from the best model (Δ AICc) and Akaike weight (wi) are shown. (DOCX) [file pone.0041671.s007.docx]

**Table S3**. AICc-based model selection based on a candidate set of models predicting large mammal species richness, aggregate mammal biomass, and a composite metric of vulnerability of species retained in 196 forest patches of the Atlantic Forest of northeastern Brazil. For each model the number of parameters (K), AIC score corrected for small sample size (AIC*_c_*), AIC*_c_* difference from the best model (Δ AIC*_c_*) and Akaike weight (w*i*) are shown.

| **Response variable** | **Model rank** | **Model** |  |  |  |  |  |  |
| --- | --- | --- | --- | --- | --- | --- | --- | --- |
|  |  |  | **LL** | **K** | **AIC_c_** | **Δ AIC_c_** | **Exp** | ***w_i_*** |
| **Species richness (S)** | 1 | PROTECT + AREA + Δ ELEV | -409.46 | 4 | 826.92 | 0.000 | 1.000 | 0.053 |
|  | 2 | PROTECT + AREA | -410.75 | 3 | 827.49 | 0.573 | 0.751 | 0.040 |
|  | 3 | PROTECT + AREA + Δ ELEV + FOREST | -409.05 | 5 | 828.09 | 1.173 | 0.556 | 0.030 |
|  | 4 | PROTECT + AREA + Δ ELEV + HPD | -409.28 | 5 | 828.57 | 1.650 | 0.438 | 0.023 |
|  | 5 | PROTECT + AREA + Δ ELEV + ELEV | -409.29 | 5 | 828.58 | 1.657 | 0.437 | 0.023 |
|  | 6 | PROTECT + AREA + Δ ELEV + RIVER | -409.29 | 5 | 828.58 | 1.661 | 0.436 | 0.023 |
|  | 7 | PROTECT + AREA + Δ ELEV + ROAD | -409.34 | 5 | 828.69 | 1.769 | 0.413 | 0.022 |
|  | 8 | PROTECT + AREA + ROAD | -410.37 | 4 | 828.73 | 1.813 | 0.404 | 0.022 |
|  | 9 | PROTECT + AREA + Δ ELEV + PROX | -409.38 | 5 | 828.75 | 1.833 | 0.400 | 0.021 |
| **Aggregate biomass (Σ BM)** | 1 | PROTECT + AREA + ELEV | -158.75 | 4 | 325.49 | 0.000 | 1.000 | 0.033 |
|  | 2 | PROTECT + AREA + ELEV + ROAD | -157.80 | 5 | 325.61 | 0.114 | 0.945 | 0.031 |
|  | 3 | PROTECT + AREA + ELEV + PROX | -157.89 | 5 | 325.79 | 0.298 | 0.862 | 0.028 |
|  | 4 | PROTECT + AREA + ROAD | -159.05 | 4 | 326.09 | 0.601 | 0.740 | 0.024 |
|  | 5 | PROTECT + AREA + ELEV + ROAD + PROX | -157.06 | 6 | 326.11 | 0.619 | 0.734 | 0.024 |
|  | 6 | PROTECT + AREA + ROAD + PROX | -158.19 | 5 | 326.37 | 0.880 | 0.644 | 0.021 |
|  | 7 | PROTECT + AREA + Δ ELEV + ROAD + PROX | -157.61 | 6 | 327.21 | 1.721 | 0.423 | 0.014 |
|  | 8 | PROTECT + AREA + Δ ELEV + ROAD | -158.61 | 5 | 327.23 | 1.734 | 0.420 | 0.014 |
|  | 9 | PROTECT + AREA + ELEV + FOREST | -158.64 | 5 | 327.29 | 1.794 | 0.408 | 0.013 |
|  | 10 | PROTECT + AREA + ELEV + RIVER | -158.65 | 5 | 327.31 | 1.817 | 0.403 | 0.013 |
|  | 11 | PROTECT + AREA + Δ ELEV + ELEV | -158.72 | 5 | 327.44 | 1.950 | 0.377 | 0.012 |
|  | 12 | PROTECT + AREA + ELEV + HPD | -158.74 | 5 | 327.47 | 1.979 | 0.372 | 0.012 |
|  | 13 | PROTECT + AREA + ELEV + ROAD + FOREST | -157.74 | 6 | 327.48 | 1.984 | 0.371 | 0.012 |
| **Aggregate vulnerability Σ (HP * 1/λ)** | 1 | PROTECT + AREA + Δ ELEV | 68.99 | 4 | -129.97 | 0.000 | 1.000 | 0.065 |
|  | 2 | PROTECT + AREA + Δ ELEV + HPD | 69.46 | 5 | -128.92 | 1.053 | 0.591 | 0.039 |
|  | 3 | PROTECT + AREA + Δ ELEV + FOREST | 69.38 | 5 | -128.76 | 1.218 | 0.544 | 0.035 |
|  | 4 | PROTECT + AREA + Δ ELEV + ROAD | 69.23 | 5 | -128.45 | 1.521 | 0.467 | 0.030 |
|  | 5 | PROTECT + AREA + Δ ELEV + PROX | 69.17 | 5 | -128.34 | 1.637 | 0.441 | 0.029 |
|  | 6 | PROTECT + AREA + Δ ELEV + RIVER | 69.05 | 5 | -128.10 | 1.872 | 0.392 | 0.026 |
|  | 7 | PROTECT + AREA + Δ ELEV + ELEV | 68.99 | 5 | -127.98 | 1.994 | 0.369 | 0.024 |
