# Supplementary material for: Pervasive Defaunation of Forest Remnants in a Tropical Biodiversity Hotspot
Source: PLoS One. 2012 Aug 14;7(8):e41671. doi: 10.1371/journal.pone.0041671 (PMC3419225; doi:10.1371/journal.pone.0041671)
Supplement: Table S4 — Averaged coefficient estimates, unconditional standard errors, unconditional variance, and relative importance of averaged coefficients calculated for aggregate properties of mammal assemblages retained within 196 forest patches of the Brazilian Atlantic forest over all models retained in the final candidate set. (DOCX) [file pone.0041671.s008.docx]

**Table S4**. Averaged coefficient estimates, unconditional standard errors, unconditional variance, and relative importance of averaged coefficients calculated for aggregate properties of mammal assemblages retained within 196 forest patches of the Brazilian Atlantic forest over all models retained in the final candidate set.

| Predictors | *β* | SE^a^ | Variance^a^ | No. models^b^ | Σ ***w_i_*** |
| --- | --- | --- | --- | --- | --- |
| **Species richness** |  |  |  |  |  |
| Protection status | 7.874 | 0.153 | 1.17E+0 | 240 | 1.000 |
| Patch area (ha) | 0.513 | 0.046 | 4.37E-2 | 126 | 0.858 |
| Elevation gradient (Δ m) | 0.003 | 2.85E-4 | 1.47E-6 | 120 | 0.575 |
| Matrix forest cover (%) | -0.007 | 0.002 | 1.38E-4 | 115 | 0.348 |
| Distance to nearest river (m) | -0.010 | 0.001 | 1.87E-5 | 115 | 0.321 |
| Between-patch proximity | 0.124 | 0.021 | 1.56E-2 | 116 | 0.317 |
| Distance to nearest road (m) | 0.004 | 0.001 | 1.18E-5 | 114 | 0.313 |
| Household density (/km^2^) | 0.006 | 0.001 | 1.15E-5 | 115 | 0.307 |
| Mean elevation (masl) | -1.53E-4 | 4.18E-5 | 5.00E-8 | 114 | 0.305 |
| **Aggregate species biomass** |  |  |  |  |  |
| Protection status | 0.774 | 0.256 | 1.38E-1 | 240 | 0.962 |
| Patch area (ha) | 0.116 | 0.051 | 7.02E-2 | 223 | 0.761 |
| Mean elevation (masl) | 1.84E-4 | 9.02E-5 | 2.28E-3 | 138 | 0.610 |
| Distance to nearest road (m) | 0.003 | 0.001 | 5.07E-9 | 124 | 0.514 |
| Between-patch proximity | -0.077 | 0.036 | 8.57E-7 | 123 | 0.443 |
| Matrix forest cover (%) | 0.005 | 0.002 | 1.13E-3 | 111 | 0.349 |
| Elevation gradient (Δ m) | 4.80E-4 | 2.08E-4 | 9.20E-6 | 112 | 0.347 |
| Distance to nearest river (m) | -0.001 | 0.001 | 5.73E-8 | 110 | 0.294 |
| Household density (/km^2^) | 2.95E-4 | 0.001 | 8.99E-7 | 103 | 0.273 |
| **Aggregate species vulnerability** |  |  |  |  |  |
| Protection status (0/1) | 0.375 | 0.082 | 7.12E-3 | 240 | 1.000 |
| Patch area (ha) | 0.029 | 0.016 | 2.03E-4 | 128 | 0.913 |
| Elevation gradient (Δ m) | 2.54E-4 | 1.21E-4 | 9.22E-9 | 126 | 0.702 |
| Household density (/km^2^) | 0.001 | 3.30E-4 | 1.21E-7 | 116 | 0.377 |
| Distance to nearest road (m) | 4.29E-4 | 2.07E-4 | 4.85E-8 | 116 | 0.344 |
| Matrix forest cover (%) | 0.001 | 0.001 | 8.12E-7 | 125 | 0.338 |
| Mean elevation (masl) | 2.58E-5 | 0.000 | 3.27E-10 | 119 | 0.307 |
| Between-patch proximity | 0.017 | 0.007 | 1.23E-4 | 125 | 0.306 |
| Distance to nearest river (m) | -0.001 | 2.83E-4 | 1.13E-7 | 118 | 0.291 |

^a^ Represent the unconditional standard error (SE) and unconditional variance of averaged coefficients.

^b^ Number of models containing each predictor variable over all models retained in the final candidate set.
